# Data on RNA-seq analysis of the oviducts of five closely related species genus *Littorina* (Mollusca, Caenogastropoda): *L. saxatilis, L. arcana, L. compressa, L. obtusata, L. fabalis*

**DOI:** 10.1016/j.dib.2022.108122

**Published:** 2022-03-31

**Authors:** Arseniy A. Lobov, Lavrentii G. Danilov, Alexey E. Masharskiy, Alexander V. Predeus, Natalia A. Mikhailova, Andrei I. Granovitch, Arina L. Maltseva

**Affiliations:** a Saint-Petersburg State University, Saint Petersburg, Russia; b Institute of Cytology of the Russian Academy of Science, Saint Petersburg, Russia; c Bioinformatics Institute, Saint Petersburg, Russia

**Keywords:** *Littorina*, *L. saxatilis*, *L. obtusata*, RNA-seq, Ovoviviparity, Mollusca, Reproductive proteins

## Abstract

In the evolution of invertebrates, the transition from egg-layers to brooders occurred many times. However, the molecular mechanisms underlying this transition are still not well understood. Recently diverged species genus *Littorina* (Mollusca, Gastropoda, Caenogastropoda, Littorinimorpha): *Littorina saxatilis, L. arcana, L. compressa, L. obtusata* and *L. fabalis* might be a fruitful model for elucidation of these mechanisms. All five species sympatrically inhabit an intertidal zone. Only *L. saxatilis* is ovoviviparous while the other four species form clutches. Although in *L. saxatilis* jelly gland of the pallial oviduct function as a brood pouch, it is not deeply modified at the morphological level in comparison to egg-laying relatives. Comparative analysis of transcriptomic profiles of the pallial oviducts of these closely related species might help to uncover the molecular mechanisms of the egg-laying to brooding transition. Unraveling of the mechanisms underlying this transition in *L. saxatilis* is important not only in aspects of reproduction biology and strategy, but also in a broader view as an example of relatively fast evolutionary transformations. We generated an RNA-seq dataset (224 104 446 clean reads) for oviducts of five species genus *Littorina*. Libraries of all five species were sequenced using Illumina HiSeq 2500; additional reads for *L. arcana* were obtained using Illumina NovaSeq 6000. Transcriptomic profiles were analyzed in pooled samples (of three individuals) with two biological replicates for each species (each biological replicate was prepared and sequenced as a separate library). The transcriptome was assembled *de novo* and annotated with five assembles corresponding to each species. The raw data were uploaded to the SRA database, the BioProject IDs are PRJNA662103 (“obtusata” group) and PRJNA707549 (“saxatilis” group).

## Specifications Table

**Table:** 

Subject	Zoology Reproductive biology
Specific subject area	Transcriptomics on pallial oviducts of the Molluscan closely related species
Type of data	Table Figures Text file
How data were acquired	Illumina HiSeq 2500, NovaSeq 6000
Data format	Raw (FASTQ) Analyzed (FASTA)
Parameters for data collection	RNA-seq by Illumina HiSeq™ 2500 of the poly(A)-RNA libraries from oviducts of *L. saxatilis, L. arcana, L. compressa, L. obtusata* and *L. fabalis* and additionally by NovaSeq 6000 for *L. arcana.*
Description of data collection	Periwinkles were collected from the wild population of the gravel-stony shores of the Varangerfjord (Barents Sea). After species identification, oviducts were excised, rinsed in filtered marine water and fixed by TRIzol reagent. Total RNA was isolated by the standard phenol-chloroform extraction [1]. Poly(A)-fraction was used for cDNA-libraries preparation with the NEBNext RNA Library Prep Kit. The libraries were sequenced by either Illumina HiSeq™ 2500 and/or NovaSeq 6000.
Data source location	City/Town/Region: Varangerfjord Country: Norway Latitude and longitude and GPS coordinates for collected samples/data: 70.063201, 29.932525 (70°03′47.5"N 29°55′57.1″E)
Data accessibility	**Repository name: NCBI Sequence Read Archive (SRA)** **Data identification number:** **BioProjectID: PRJNA707549** **BioProjectID: PRJNA662103** **Instructions for accessing these data:** **The raw sequence reads can be accessed via NCBI SRA with BioProjectID: PRJNA707549** **Direct link to the data**: https://www.ncbi.nlm.nih.gov/bioproject/PRJNA707549 **BioProjectID: PRJNA662103** **Direct link to the data**: https://www.ncbi.nlm.nih.gov/bioproject/PRJNA662103

## Value of the Data

• The data represent the transcriptomic dataset of reproductive tissues of several recently diverged gastropod species pursuing different reproductive strategies. Such evolutionary transition is expected to be accompanied by rapid divergence of the specific groups of genes associated with the immune system, reproduction and development. Thus, our dataset may be informative for a wide range of specialists in evolutionary biology and contiguous areas.
• The dataset displays genes that are expressed in pallial oviducts of gastropods with two different reproductive strategies. The data may be useful for specialists in the reproductive biology of invertebrates investigating fundamental aspects of sexual reproduction and for malacologists.
• The dataset can be used for CDS-prediction during analysis of the Molluscan genomes, search and analysis of “orphan” genes, analysis of evolution of specific target protein groups and for specific molecular analysis, e.g. characterization of target transcripts expression patterns by *in situ* RNA-hybridisation.

## 1 Data Description

Comparative morphology of different reproductive systems has actively developed in the last centuries. Nevertheless, the molecular background of reproduction of invertebrates has been investigated only in several model objects. Particularly, the transition from egg-layers to brooders has been investigated in many invertebrate taxa at the morphological level, but molecular mechanisms responsible for such transition are still poorly investigated. From this point of view, recently diverged species genus *Littorina* (Mollusca, Gastropoda, Caenogastropoda, Littorinimorpha) seem to be a fruitful model for elucidation of these mechanisms.

At the Europian gravel-stony shores, periwinkles genus *Littorina* Férussac, 1822 subgenus *Neritrema* Récluz, 1869 include two groups of closely related species: “saxatilis” group (*Littorina saxatilis* (Olivi 1792), *L. arcana* Hannaford Ellis 1978 and *L. compressa* Jeffreys 1865) and “obtusata” group (*L. obtusata* (Linnaeus 1758), *L. fabalis* (Turton 1825)).

These species are among the most common inhabitants of the Northern Atlantic European seashores and are routinely used as a model to analyze anatomy, physiology and morphology of gastropods. Besides, they are an informative model for evolutionary ecology, especially *L. saxatilis* [2,3]. Particularly, differences in reproductive strategies and anatomy of reproductive system of the *Neritrema* species are well described [2]. Four of them form clutches and only *L. saxatilis* has shifted to ovoviviparity. This transition of *L. saxatilis* is associated with anatomical changes in the pallial oviduct: the jelly gland of the pallial oviduct function as a brood pouch. Neverheless, pallial oviduct has not deeply modified at the morphological level in comparison to egg-laying relatives, and the existence of physiological and biochemical changes, such as secretion of specific proteins and shifts in the immune system functioning, is quite expectable. Thus, the comparison based on ‘omics’-technologies between pallial oviducts of *L. saxatilis* and four other species may help to unravel the mechanisms underlying the egg-laying to brooding transition.

The genome of *L. saxatilis* has been published, and several tissue transcriptomes of the *Neritrema* species are available now [3,4]. Nevertheless, the transcriptomes of the pallial oviducts of closely related European *Neritrema* species have not been sequenced yet.

Here we present the RNA-seq raw reads and transcriptomes *de novo* assembled for the oviducts of five species genus *Littorina: L. saxatilis, L. arcana, L. compressa, L. obtusata* and *L. fabalis*. To reduce intragroup biological dispersion, we used pooled samples [5] – each biological replicate consisted of material from three individuals.

The raw data are stored in the NCBI database. We deposited five BioSamples corresponding to the five *Neritrema* species with two SRA experiments per each BioSample corresponding to the two biological replicates obtained per each species. BioSamples were separated to two BioProjects corresponding to “obtusata” (PRJNA662103) and “saxatilis” (PRJNA707549) groups of closely related species. The basic statistics and accession numbers for each file are in Table 1.

**Table 1:** Resulted statistics and accession numbers of *Littorina* sp. oviducts assemblies.

Species	BioProject accession number	Number of unigenes	Total unigenes lenghts, bp	Average unigenes lenghts, bp	Total number of clean reads	BioSample accession number	Biological replicate	SRA accession number	Instrument
*L. saxatilis*	PRJNA707549	86 329	9 037	714	23 941 141	SAMN18209702	1	SRR13962427	HiSeq 2500
							2	SRR13962426	HiSeq 2500
*L. arcana*		80 695	14 680	715.8	39 074 348	SAMN18209703	1	SRR13962425	HiSeq 2500
							2	SRR13962424	NovoSeq 6000
*L. compressa*		45 182	20 682	1158.8	58 007 281	SAMN18209704	1	SRR13962423	HiSeq 2500
							2	SRR13962422	HiSeq 2500
*L. obtusata*	PRJNA662103	99 513	10 822	720.6	46 098 082	SAMN16076810	1	SRR12605103	HiSeq 2500
							2	SRR12605102	HiSeq 2500
*L. fabalis*		58 055	11 253	680.1	56 983 594	SAMN16076809	1	SRR12605105	HiSeq 2500
							2	SRR12605104	HiSeq 2500
				Total number	224 104 446				

**Fig. 1:**
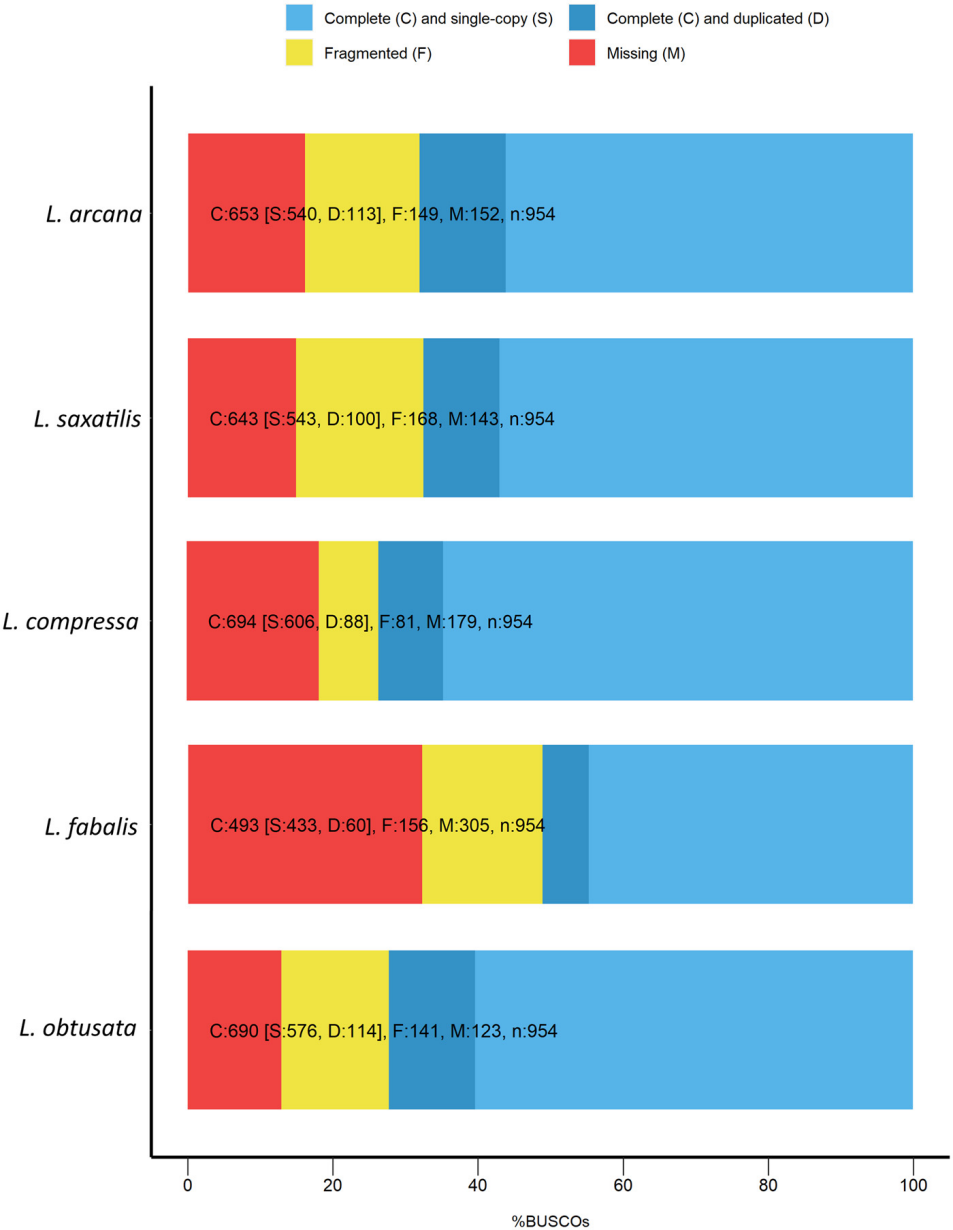
Bar chart demonstrates the completeness of assemblies of the oviduct transcriptomes of six species genus *Littorina* based on the BUSCO analysis against the Metazoa database.

The quality and completeness of obtained assemblies was estimated by the BUSCO analysis against the Metazoa database. Assemblies for all species have less than 30% of missed genes (Fig. 1).

**Fig. 2:**
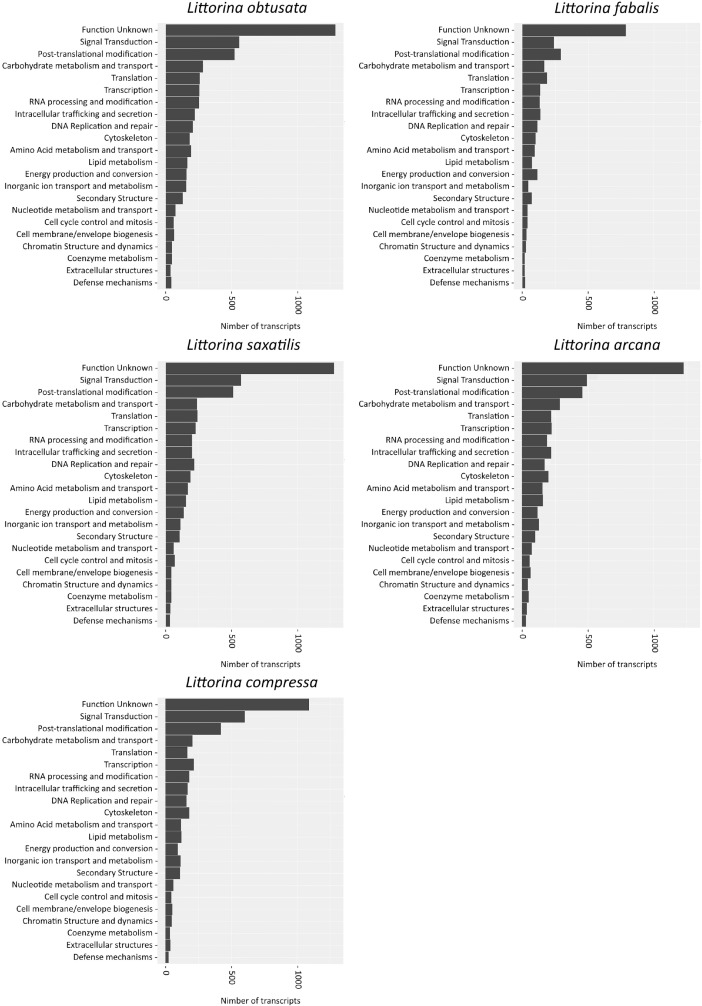
top-22 categories of Clusters of Orthologous Groups (COGs) in oviduct transcriptomes of five *Neritrema* species.

For the functional annotation of the assemblies, we mapped contigs against the database of Clusters of Orthologous Groups of proteins (COGs) within the eggNOG-mapper. The oviduct transcriptomes of all species had a similar distribution pattern of the orthologous groups, with the «Function Unknown» as the most abundant category (Fig. 2).

## 2 Experimental Design, Materials and Methods

### 2.1 Animals and tissue preparation

Females of *L. saxatilis, L. arcana, L. compressa, L. obtusata* and *L. fabalis* were collected from the wild populations at the Varangerfjord gravel-stony shores near Vads*ø* (70°03′47.5"N 29°55′57.1"E) and transported to the laboratory. The snails were dissected no longer than 8 h after collection for the species identification according to [2,6]. The oviducts including receptacle were cut out and rinsed twice in filtered marine water. In case of *L. saxatilis,* the embryos were removed from the brood pouch before rinsing. Then the oviducts were cut into fragments several mm in diameter and fixed with 1 ml of TRIzol (Ambion). The samples in TRIzol were transferred to the laboratory under -20°C conditions and then stored at -80°C. Tissues from three individuals were pooled; two biological replicates were prepared for each species and analyzed as separate libraries (Table. 1).

### 2.2 cDNA library preparation and high-throughput sequencing

The tissues were mechanically homogenized and total RNA was isolated according to the standard protocol of TRIzol extraction [1]. The quality of RNA was tested by agarose and capillary electrophoresis using QIAxcel Advanced (QIAGEN, Germany). We used only RNA with the RNA integrity score (RIS) higher than 5. 500 ng of RNA of each sample was used for the isolation of poly(A)-fraction using NEBNext® Poly(A) mRNA Magnetic Isolation Module according to manufacturer recommendations; then the RNA was quantified by Qubit fluorometer (Invitrogen, USA) and used for library preparation using NEBNext® UltraTM Directional RNA Library Prep Kit for Illumina® with NEBNext® Multiplex Oligos for Illumina® (Dual Index Primers Set 1) according to the manufacturer recommendations (https://international.neb.com/products/e7420-nebnext-ultra-directional-rna-library-prep-kit-for-illumina#Protocols,%20Manuals%20&%20Usage; accessed 17.08.2021). The quality of libraries was tested by capillary electrophoresis using QIAxcel Advanced (QIAGEN, Germany). The peak lengths of the analyzed libraries were varying from 296 to 378 bp.

Library preparation and sequencing were performed in St. Petersburg State University “Biobank” core facility (St. Petersburg, Russia) using Illumina HiSeq2500 and in the commercial service provider “Evrogen” (Moscow, Russia) using Illumina NovaSeq 6000 (“Evrogen”); 150-bp paired-end (PE) sequencing was used.

All samples were analysed in the same cell by Illumina HiSeq 2500. The second biological replicate of *L. arcana* (prepared with the same Library Prep Kit) was obtained using NovaSeq 6000, as HiSeq2500-run brought low reads number in this sample. Since it possibly could lead to some bias during quantitative analysis, this sample data should be used with care. However, HiSeq 2500 and NovaSeq 6000 have similar error rates [7] and our data is fully appropriate for any qualitative comparative analysis, mass spectrometric protein identification, and other non-quantitative analytical purposes.

### 2.3 Data filtering

The quality of the sequencing output was assessed using the FastQC software [8]. Adapters were removed using cutadapt v3.2 [9]; sequences were trimmed and filtered with the Trimmomatic v0.39 software with command trimmomatic-0.39.jar PE -threads 6 [seq_name_1 seq_name_2] seq_name_1_unpaired.fastq seq_name_1_paired. seq_name_2_unpaired.fastq seq_name_2_paired ILLUMINACLIP:Trimmomatic-0.39/adapters/TruSeq3-PE-2.fa:2:30:10:2:TRUE SLIDINGWINDOW:4:20 MAXINFO:50:0.8 MINLEN:25 [10].

### 2.4 De novo transcriptome assembly

Trinity RNA-Seq assembly software package version 2.9.1 [11] with the command “Trinity –seqType fq–max_memory 80G –left [LEFT_READS_FILES] –right [RIGHT_READ_FILES] –CPU 20 –min_contig_length 200 –super_transcripts –full_cleanup” was used to assemble *de novo* all the transcriptomes without a reference genome. Next, we used CD-HIT-est [12] to cluster similar sequences (with a comand cdhit-est -i [input_file_name] -o [output_file_name] -c 0.95 -d 0 -g 1 -r 1: with a 95% similarity rate) and Transrate v1.0.1 [13] to improve the quality of the transcriptome assembly. Transcriptome completeness was assessed using BUSCO 4.2 [14] against the Metazoa Odb10 BUSCO dataset with –evalue 1e-3. To predict the coding sequences, we used the script TransDecoder.LongOrfs [15], the minimum protein size was taken as 100 amino acids. Finally, the transcriptomes were filtered with 250 bp minimal transcript lengths and annotated with the eggNOG-mapper (accessed by 01.03.2021, http://eggnog-mapper.embl.de/) [16].

## Ethics Statement

All experiments with specimens of the genus *Littorina* were performed in compliance with the ARRIVE guidelines and were carried out in accordance with the U.K. Animals (Scientific Procedures) Act, 1986 and EU Directive 2010/63/EU for animal experiments.

## CRediT authorship contribution statement

**Arseniy A. Lobov:** Investigation, Visualization, Writing – original draft, Data curation. **Lavrentii G. Danilov:** Formal analysis, Software. **Alexey E. Masharskiy:** Investigation. **Alexander V. Predeus:** Formal analysis. **Natalia A. Mikhailova:** Project administration. **Andrei I. Granovitch:** Supervision, Resources. **Arina L. Maltseva:** Funding acquisition, Writing – review & editing, Supervision, Conceptualization.

## Declaration of Competing Interest

The authors declare that they have no known competing financial interests or personal relationships which have or could be perceived to have influenced the work reported in this article.

## Data Availability

Species-specific proteins in the oviducts of sibling species: proteotranscriptomic study of Littorina fabalis and L. obtusata (Original data) (NCBI). RNA-seq of oviduct transcriptomes of three species of the ``saxatilis'' group of closely related molluscs species: Littorina saxatilis, L. compressa, L. arcana (Original data) (NCBI).
